# A green chemistry approach for synthesizing biocompatible gold nanoparticles

**DOI:** 10.1186/1556-276X-9-248

**Published:** 2014-05-21

**Authors:** Sangiliyandi Gurunathan, JaeWoong Han, Jung Hyun Park, Jin-Hoi Kim

**Affiliations:** 1 Department of Animal Biotechnology, Konkuk University, 1 Hwayang-Dong, Gwangin-gu, Seoul 143-701, South Korea; 2 GS Institute of Bio and Nanotechnology, Coimbatore, Tamilnadu, India

**Keywords:** AuNPs, Biocompatibility, *Ganoderma* spp, Human breast cancer cells, Transmission electron microscopy, UV-visible spectroscopy

## Abstract

Gold nanoparticles (AuNPs) are a fascinating class of nanomaterial that can be used for a wide range of biomedical applications, including bio-imaging, lateral flow assays, environmental detection and purification, data storage, drug delivery, biomarkers, catalysis, chemical sensors, and DNA detection. Biological synthesis of nanoparticles appears to be simple, cost-effective, non-toxic, and easy to use for controlling size, shape, and stability, which is unlike the chemically synthesized nanoparticles. The aim of this study was to synthesize homogeneous AuNPs using pharmaceutically important *Ganoderma* spp*.* We developed a simple, non-toxic, and green method for water-soluble AuNP synthesis by treating gold (III) chloride trihydrate (HAuCl_4_) with a hot aqueous extract of the *Ganoderma* spp*.* mycelia. The formation of biologically synthesized AuNPs (bio-AuNPs) was characterized by ultraviolet (UV)-visible absorption spectroscopy, X-ray diffraction (XRD), Fourier transform infrared spectroscopy (FTIR), energy dispersive X-ray (EDX), dynamic light scattering (DLS), and transmission electron microscopy (TEM). Furthermore, the biocompatibility of as-prepared AuNPs was evaluated using a series of assays, such as cell viability, lactate dehydrogenase leakage, and reactive oxygen species generation (ROS) in human breast cancer cells (MDA-MB-231). The color change of the solution from yellow to reddish pink and strong surface plasmon resonance were observed at 520 nm using UV-visible spectroscopy, and that indicated the formation of AuNPs. DLS analysis revealed the size distribution of AuNPs in liquid solution, and the average size of AuNPs was 20 nm. The size and morphology of AuNPs were investigated using TEM. The biocompatibility effect of as-prepared AuNPs was investigated in MDA-MB-231 breast cancer cells by using various concentrations of AuNPs (10 to 100 μM) for 24 h. Our findings suggest that AuNPs are non-cytotoxic and biocompatible. To the best of our knowledge, this is the first report to describe the synthesis of monodispersed, biocompatible, and soluble AuNPs with an average size of 20 nm using *Ganoderma* spp. This study opens up new possibilities of using an inexpensive and non-toxic mushroom extract as a reducing and stabilizing agent for the synthesis of size-controlled, large-scale, biocompatible, and monodispersed AuNPs, which may have future diagnostic and therapeutic applications.

## Background

In recent years, gold nanoparticles (AuNPs) have been of great research interest because of their unique properties, such as size- and shape-dependent optoelectronic, physiochemical, and biological properties as well as various potential therapeutic applications. AuNPs possess distinct physical and chemical properties that make them excellent tools for creating novel chemical and biological sensors [1-3]. First, AuNPs can be synthesized using a simple method and made highly stable. Second, they possess unique optoelectronic properties. Third, they provide high surface-to-volume ratios with excellent biocompatibility when using appropriate ligands [1]. Fourth, these AuNP properties can be readily tuned by varying their size and shape as well as the surrounding chemical environment [3].

Because of their stability, oxidation resistance, and biocompatibility, AuNPs have a wide range of potential applications, such as in electronics and photonics, catalysis, information storage, chemical sensing and imaging, drug delivery, and biological labeling [4,5]. The tuning of AuNPs is an important process to enhance versatility in defining and controlling the shape [5]. Thus, new methodologies are essential for designing shape-controlled synthesis of AuNPs [6-8].

Several synthetic chemical methods have been adopted for AuNP synthesis, including physical methods, such as attrition and pyrolysis, which were previously utilized for the synthesis of metallic nanoparticles [9]. Alternatively, chemical methods are the most widely and traditionally used methods and incorporate various reducing agents, such as hydrazine [9] and sodium borohydride [10]. However, many of these methods can be cumbersome and involve the use of toxic chemicals, high temperatures, and pressures and, most importantly, can cause the particles to become unstable or aggregate upon interaction with biological media or biomolecules [11]. At the same time, these approaches produce multi-shaped nanoparticles that require purification by differential centrifugation, and consequently have a low yield [12,13]. Therefore, there is a constant need for new methodologies to produce shape-controlled synthesis of AuNPs.

Recently, several labs have been interested in developing methodologies for synthesis of nanomaterials using a green chemistry approach, which is an alternate approach to biosynthesizing nanomaterials that relies on natural organisms for the reduction of metal ions into stable nanocrystals [14-21]. Biological methods are supposed to yield novel and complex structural entities, unlike those obtained using conventional techniques [14,15,22]. A number of microbial species have been used for synthesis of metal nanoparticles but without much success in achieving shape control. The shape-controlled microbial synthesis of nanostructures is an exciting new area with considerable potential for development. Recently, Das et al. reported the synthesis of single-crystalline AuNPs [19] and different nanostructures from HAuCl_4_ using *Rhizopus oryzae*[5].

Biological methods exhibit size and shape control over a diverse array of materials, and they also facilitate mass production, high yield, and reproducibility [23,24]. Biosynthesis of AuNPs and silver nanoparticles (AgNPs) have been reported in different prokaryotic organisms, including *Bacillus licheniformis*[20]*, Brevibacterium casei*[21]*, Bacillus subtilis*[25]*, Escherichia coli*[26]*, Lactobacillus*[27]*, Pseudomonas aeruginosa*[28]*,* and *Rhodopseudomonas capsulate*[29]. Several researchers exploited fungi as reducing agents for AgNP synthesis, including fungi such as *Verticillium*[14], *Fusarium oxysporum*[16]*, Aspergillus fumigatus*[30]*, Penicillium fellutanum*[31]*, Volvariella volvacea*[32]*, Pleurotus florida*[33]*, Candida*[34], *Ganoderma lucidum*[35], and *Neurospora crassa*[36].

Among nanoparticles, AuNPs have immense potential for cancer diagnosis and therapy. Conjugation of AuNPs to ligands on cancer cells allows molecular imaging and detection of cancer [37]. Further, AuNPs have potential applications in electronics, catalysis, biological sensors, cancer diagnostics, therapeutics, nanomedicine, and environmental work, because they have several merits, such as the fact that they are easy to synthesize, cost effective, and non-toxic, and they have easy functionalization, optical properties, facile surface chemistry, and biocompatibility [37,38]. Moreover, biological processes could provide significant yield and are free from downstream processing; therefore, many researchers are interested in synthesizing nanoparticles with green manufacturing technology that uses bacteria, fungi, plants, and plant products.

In most studies, either AuNPs or AgNPs were synthesized using bacteria. Many fungi have not been explored, including those mentioned above, and only a few fungi have been investigated for AuNP and AgNP synthesis. Among fungi that have not been tested, *Ganoderma* spp. have long been used as medicinal mushrooms in Asia, and they have an array of pharmacological properties, including immunomodulatory activity and pharmacological properties [39]. *Ganoderma* spp. have several advantages: They have large quantities of viable mycelia, they are cost-effective, there is easy downstream processing, and they are non-pathogenic. Moreover, the synthesized AuNPs are highly soluble in water. Therefore, the aim of this study was to investigate the possible use of *Ganoderma* spp. as green producers for AuNP synthesis and to further evaluate the biocompatibility effect of as-prepared AuNPs in human breast cancer cells (MDA-MB-231).

## Methods

### Reagents

Gold (III) chloride trihydrate was purchased from Sigma (St. Louis, MO, USA). Penicillin-streptomycin solution, trypsin-EDTA solution, Dulbecco's modified Eagle's medium (DMEM/F-12), and 1% antibiotic-antimycotic solution were obtained from Life Technologies GIBCO (Grand Island, NY, USA). All the other chemicals and reagents were purchased from Sigma (St. Louis, MO, USA), unless otherwise specified.

### Culturing and maintenance of *Ganoderma* spp

The culture of *Ganoderma* spp. was collected from a tropical forest near Pollachi, Tamilnadu, India. Culturing and maintenance were conducted as described in previous studies, with suitable modifications [40,41]. Briefly, the mycelia were cultured on potato dextrose agar (PDA) and incubated at 28°C ± 2°C for 7 days. The mycelia were then transferred to glucose yeast malt peptone broth (GYMP). The inoculated medium was incubated at 28°C ± 2°C and agitated at 150 rpm for 10 days. After incubation, the mycelia were harvested, washed with distilled water, freeze-dried, and stored at 4°C in air-tight containers, prior to use.

### Preparation of mycelia hot aqueous extract

The preparation of mushroom extract was carried out according to a method described in previous studies [40,41], with suitable modifications. In brief, the freeze-dried mycelia were soaked in distilled water at a ratio of 1:20 and double boiled for 45 min, left to cool, and filtered through Whatman filter paper No. 4. The hot aqueous extract was then freeze-dried at -70°C ± 2°C for 48 h and stored at 4°C in airtight containers. The freeze-dried hot aqueous extract of the mycelia was used as the reducing and stabilizing agent for AuNP synthesis.

### Synthesis of AuNPs

Synthesis of AuNPs was carried out according to the method described earlier [21]. In a typical reaction, 1 mg/mL of freeze-dried hot aqueous mushroom mycelia extract was mixed with an aqueous solution of 1 mM HAuCl_4_ solution and kept at room temperature for 24 h. Synthesis was observed using ultraviolet (UV)-visible spectroscopy. The color change observed was from pale yellow to purple. To compare the efficiency of biologically prepared AuNPs, we used citrate-mediated synthesis of AuNPs (chem-AuNPs) from Sigma.

### Characterization of AuNPs

Characterization of synthesized AuNPs was carried out according to previously described methods [20]. The nanoparticles were primarily characterized by UV-visible spectroscopy, which has proven to be a very useful technique for nanoparticle analysis [26]. UV-visible (UV-vis) spectra were obtained using a WPA (Biowave II, Biochrom, Cambridge, UK). Dispersion particle size was measured by Zetasizer Nano ZS90 (Malvern Instruments Limited, Malvern, UK).

The synthesized AuNPs were freeze dried, powdered, and used for X-ray diffraction (XRD) analysis. The spectra were evaluated using an X-ray diffractometer (PHILIPS X'Pert-MPD diffractometer, Amsterdam, the Netherlands) and Cu-K*α* radiation (1.5405 Å) over an angular range of 10° to 80° at 40 kV and 30 mA. The dried powder was diluted with potassium bromide at a the ratio of 1:100, and the results were recorded using the Fourier transform infrared spectroscopy (FTIR; PerkinElmer Inc., Walham, MA, USA) and spectrum GX spectrometry within the range of 500 to 4,000 cm^-1^.

Transmission electron microscopy (TEM, JEM-1200EX, JEOL Ltd., Tokyo, Japan) was used to determine the size and morphology of AuNPs. AuNPs were prepared by dropping a small amount of aqueous dispersion on copper grids, which were dried and then examined in the TEM. Further, the presence of Au metals in the sample was analyzed by energy dispersive X-ray analysis (EDX) combined with a field emission SEM.

### Cell culture

MDA-MB-231 human breast cancer cells were kindly provided by Kyung Jin Lee, Institute for Life Sciences, ASAN Medical Center, University of Ulsan College of Medicine. MDA-MB-231 breast cancer cell lines were grown adherently and maintained in DMEM containing 10% fetal calf serum (FCS) and 1% antibiotic solution containing penicillin and streptomycin at 37°C under 5% CO_2_. All the experiments were performed in six-well plates, unless stated otherwise. Cells were seeded onto plates at a density of 1 × 10^6^ cells per well and incubated for 24 h prior to the experiments. The cells were washed with phosphate buffered saline (PBS, pH 7.4) and incubated in fresh medium containing different concentrations of AuNPs dissolved in water.

### Cell viability assay

In order to evaluate the biocompatibility of the as-prepared AuNPs, we carried out cell viability assay in breast cancer cells (MDA-MB-231) by using MTT reagents. In addition, to compare the biocompatibility effect of bio-AuNPs, we used chemical-mediated synthesis of chem-AuNPs as a positive control. Cell viability was measured using the 3-(4,5-dimethylthiazol-2-yl)-2,5-diphenyltetrazolium bromide dye reduction assay performed to determine the cytotoxic effect of the AuNPs at various concentrations. Briefly, the cells were plated onto 96-well flat-bottom culture plates with various concentrations of AuNPs (0 to100 μM). All the cultures were incubated for 24 h at 37°C in a humidified incubator. After 24 h of incubation (37°C, 5% CO_2_ in a humid atmosphere), 10 μL of MTT (5 mg/mL in PBS) was added to each well, and the plate was incubated for another 4 h at 37°C. The resulting formazan was dissolved in 100 μL of DMSO with gentle shaking at 37°C, and the absorbance was measured at 595 nm by using an ELISA reader (Spectra MAX; Molecular Devices, Sunnyvale, CA, USA). The results are shown as the mean of three independent experiments.

### Membrane integrity

Cell membrane integrity of MDA-MB-231 cells was evaluated by determining the activity of lactate dehydrogenase (LDH) leaking out of the cell, according to the manufacturer's instructions (*in vitro* toxicology assay kit, TOX7, Sigma, USA). The LDH assay is based on the release of the cytosolic enzyme LDH from cells with damaged cellular membranes. Thus, in cell culture, AuNPs induced cytotoxicity and were quantitatively analyzed by measuring the activity of LDH in the supernatant. Briefly, cells were exposed to various concentrations of AuNPs for 24 h, and then 100 μL per well of each cell-free supernatant was transferred in triplicates into wells in a 96-well plate, and 100 μL of LDH-assay reaction mixture was added to each well. After 3-h incubation under standard conditions, the optical density of the generated color was determined at a wavelength of 490 nm using a Microplate Reader.

### Determination of ROS

Intracellular reactive oxygen species (ROS) were measured based on the intracellular peroxide-dependent oxidation of 2′,7′-dichlorodihydrofluorescein diacetate (DCFH-DA, Molecular Probes, Eugene, OR, USA) to form the fluorescent compound 2′,7′-dichlorofluorescein (DCF), as previously described. Cells were seeded onto 24-well plates at a density of 5 × 10^4^ cells per well and cultured for 24 h. After washing twice with PBS, fresh medium containing 100 μM of AuNPs, 1 mM H_2_O_2_, or AgNPs (5 μg/mL) was added, and the cells were incubated for 24 h. For the control, cells were added to 20 μM of DCFH-DA and incubation continued for 30 min at 37°C. The cells were rinsed with PBS, 2 mL of PBS was added to each well, and fluorescence intensity was determined with a spectrofluorometer (Gemini EM) with excitation at 485 nm and emission at 530 nm. For the control, had antioxidant *N*-acetyl-l-cystein (NAC, 5 mM) was added to the cells grown in 24-well plates (for 24 h) for 1 h prior to exposure to AuNPs, 1 mM H_2_O_2_, or AgNPs (5 μg/mL) for 24 h. We then added 20 μM of DCFH-DA, and the cells were incubated for 30 min at 37°C before measuring DCF fluorescence changes as described.

## Results and discussion

### Extracellular synthesis of AuNPs

**Figure 1:**
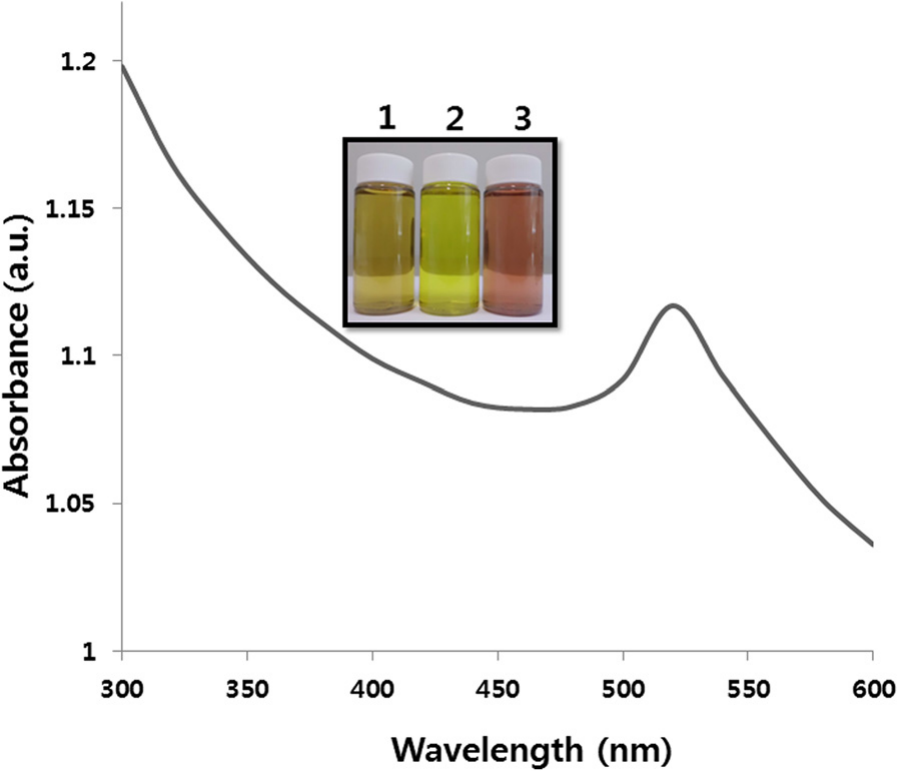
**Synthesis and characterization of AuNPs.** The figure inset shows tubes containing samples of the *Ganoderma* spp*.* extract (1); 1 mM aqueous HAuCl_4_ (2); extract after incubation with HAuCl_4_ (3). The absorption spectrum of AuNPs exhibited a strong broad peak at 520 nm, and this band was assigned to surface plasmon resonance of the particles.

Primary characterization of the ability of *Ganoderma* spp*.* mushroom extract for AuNP synthesis was analyzed. The Figure 1 inset shows tubes with the *Ganoderma* spp*.* mycelia extract [1], HAuCl_4_[2], and extract after reaction with HAuCl_4_ ions for 24 h [3]. As expected, the color changed from pale yellow to deep purple in the presence of the extract, which indicates AuNP formation and is evidence of synthesis.

Further, to determine if there was AuNP synthesis, the biosynthesis of nanoparticle reactions was monitored by UV-vis spectroscopy. UV-vis absorption spectroscopy is the most widely used technique for characterizing the optical properties and electronic structure of nanoparticles, because the absorption bands are related to the diameter and different aspect ratios of metal nanoparticles, including size and shape [42]. As shown in Figure 1, the spectra of AuNP synthesis showed a gradual increase in the surface plasmon resonance (SPR) excitation peak centered at 520 nm, which is characteristic of AuNPs [11,43]. This further indicates that the mushroom extract could be useful as a reducing agent for AuNP synthesis. Control reactions in the absence of mushroom extract exhibited no change in color or absorbance at 520 nm, clearly indicating that the protein and polysaccharides found in the extract are responsible for biosynthesis of AuNPs.

Previous studies demonstrated that metal biotransformation might involve a complex of either capping proteins/peptides and reductases, quinines, cytochromes, phytochelatins, or electron shuttles that are known to reduce various metals and metal oxides [11,43-46]. Das et al. [47] proposed possible mechanisms of AuNP synthesis in *Rhizopus oryzae*. The first mechanism is binding of Au (III) on the cell wall through electrostatic interaction followed by reduction to AuNPs by proteins/enzymes present on the cell wall, and the second is diffusion or transportation of Au (III) into the cytoplasm and protein/enzymatic reduction to form AuNPs. Taken together, these results indicate that AuNP synthesis could be facilitated by the presence of proteins in the extract.

### XRD analysis of AuNPs

**Figure 2:**
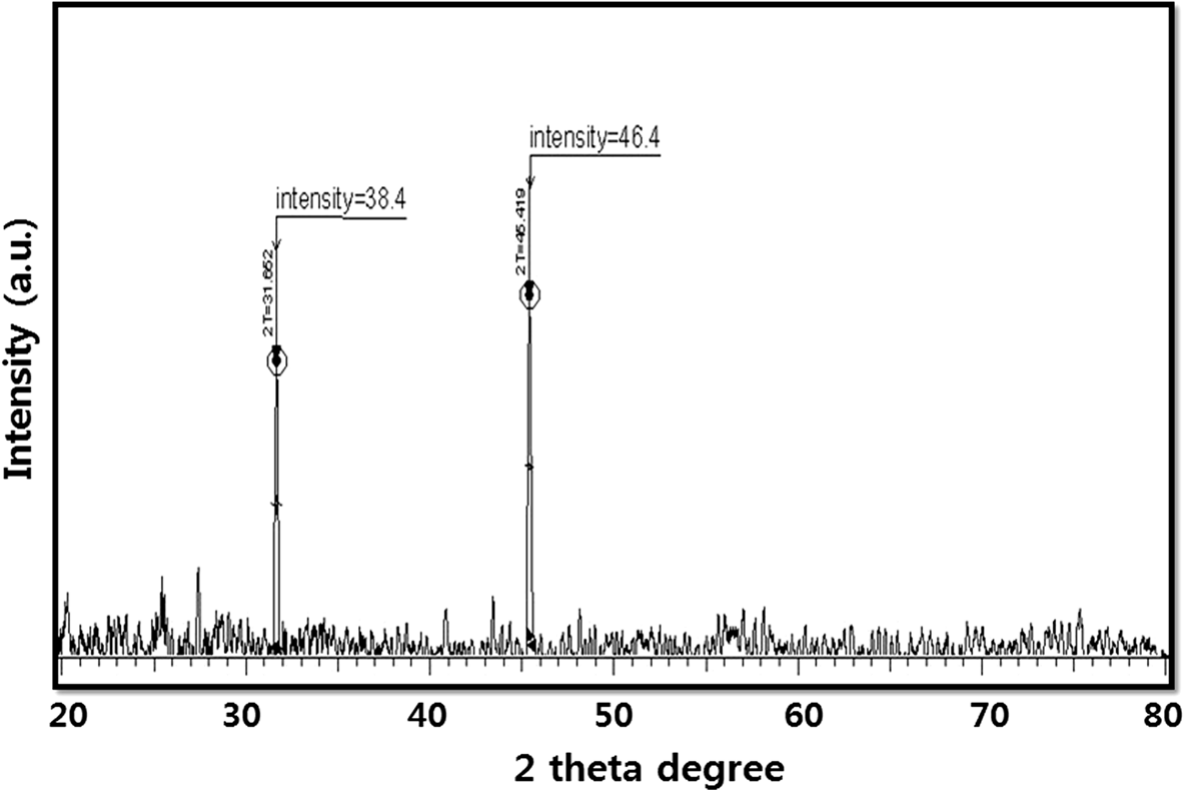
X-ray diffraction spectra of AuNPs.

The crystalline nature of as-prepared AuNPs was confirmed using XRD. The XRD spectrum shows two predominant peaks that agree with Bragg's reflection of AuNPs reported in a previous study, which used extracellular and intracellular culture supernatant of *Aspergillus fumigatus* and *Aspergillus flavus*[48]. The diffraction peaks, which appeared at 31.6°C and 45.4°C corresponded to the (111) and (200) planes, respectively (Figure 2). No extra peak was observed in the diffraction peaks, which indicates that the as-prepared AuNPs were highly purified without any contamination.

Gupta and Bector [48] observed four different intense peaks at 2*θ* angle: 38.22, 44.42, 64.71, and 77.62 with Bragg reflections corresponding to (111), (200), (220), and (311) in biomass-associated AuNPs. Alternatively, only a single prominent peak was observed at 2*θ* angle: 38.22 with a Bragg reflection corresponding to (111) in extracellular AuNPs. Our present findings are consistent with earlier studies that used biological methods to synthesize AuNPs using plant extracts [49-51], yeast [16], and bacteria [20].

### FTIR analysis

**Figure 3:**
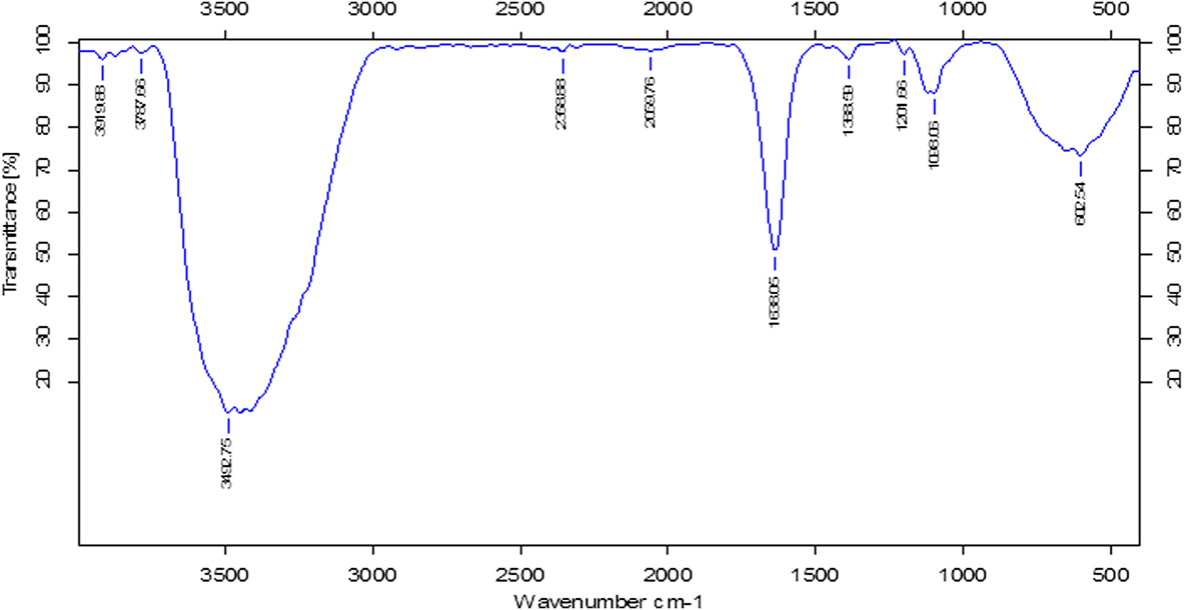
FTIR spectra of AuNPs.

The AuNPs synthesized by *Ganoderma* spp. mushroom extract were subjected to FTIR analysis to identify the biomolecules involved in stabilizing the nanoparticles in solution [51]. The AuNPs synthesized by mushroom extract yielded strong bands at 602, 1096, 1201, 1388, and 1636 cm^-1^ (Figure 3). These bands correspond to the amide I, II, and III bands of polypeptides/proteins, and are consistent with previous reports [51,52]. As suggested by Sastry et al., the polypeptides found in the mushroom extracts served as capping agents in AuNPs, particularly glutathione, which is known to be produced by yeast cells [53].

It is well known that proteins can bind to AuNPs either through free amine groups or cysteine residues in the proteins [54]; therefore, stabilization of the AuNPs by surface-bound proteins is a possibility in the case of AuNPs synthesized by *Ganoderma* spp. Additionally, the bands at 1,636 cm^-1^ can be assigned to the vibrational modes of C=C double bonds of these molecules. The large peak between 1,500 and 1,700 cm^-1^ falls in the region of C=O stretching frequency, and the bands at 3,492 cm^-1^ correspond to carbonyl and hydroxyl functional groups in alcohols and phenol derivatives [11,16,55]. The FTIR results show that the surface capping of AuNPs synthesized by the mushroom extract is predominantly by proteins. Moreover, our results are consistent with those reported earlier for biosynthesized nanoparticles [11,16,50,51,55].

**Figure 4:**
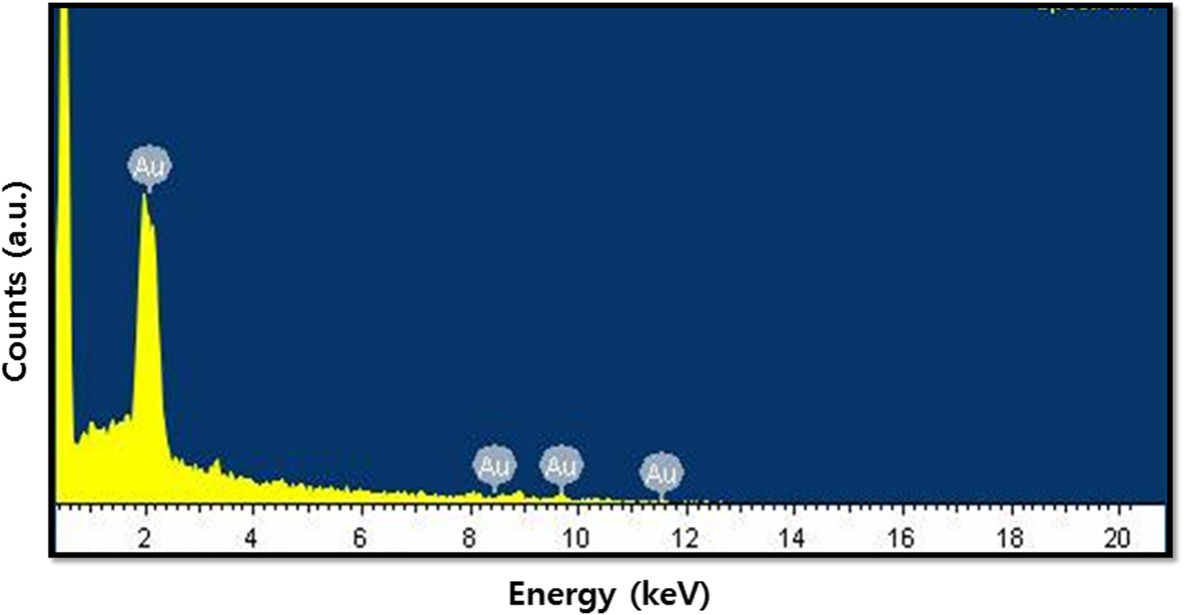
EDX spectra of AuNPs.

AuNP synthesis by the *Ganoderma* spp. extract was confirmed using EDS and spectra, as represented in Figure 4. The EDS profile shows a strong gold signal along with weak oxygen and carbon peaks, which may have originated from the biomolecules of the mushroom extract that bound to the AuNP surfaces.

### Particle size analysis

**Figure 5:**
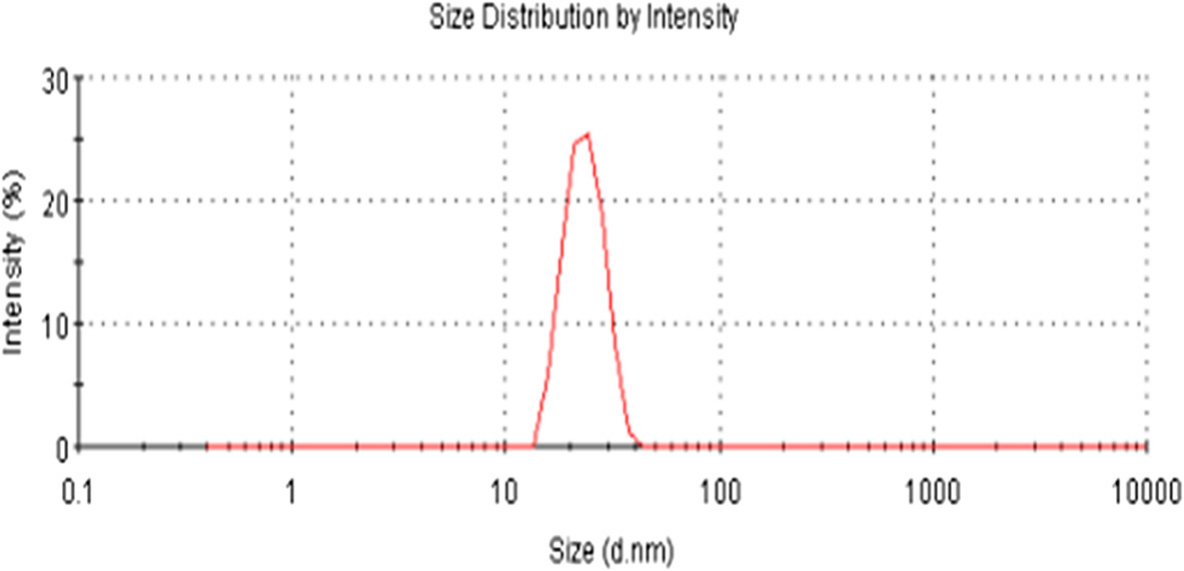
**Size distribution analysis of AuNPs by DLS.** The particle-size distribution revealed that the average particle size was 20 nm.

Further characterization was carried out to determine the particle size distributions using dynamic light scattering (DLS) technique, which reveals the average hydrodynamic diameter of particles in a liquid suspension. These particle sizes are well within the range reported for photoluminescence of AuNPs [15]. Figure 5 shows the DLS analysis of mushroom extract-mediated synthesis of AuNPs; the average size (20 nm) is within the expected range of particle sizes between 15 to 30 nm and is very similar to the size that was observed in TEM (20 nm). However, for particle sizes larger than 25 nm, the bandwidth increases with the increase in particle size [42], and nanoparticles such as gold and silver have also been shown to exhibit size-dependent optical properties. Husseiny et al. [28] observed the absorption spectra of AuNPs using three different strains of *P. aeruginosa* ATCC 90271, *P. aeruginosa*, and *P. aeruginosa*, and the maximum absorption peaks observed were 543, 540, and 531 nm corresponding to particle sizes of 30 ± 10, 25 ± 15, and 15 ± 5 nm, respectively.

In our results, we found that the absorption peak is at 520 nm, which corresponds to a particle size of 20 nm. Moreover, our results are consistent with the absorption spectra and particle size analysis data obtained for chemically prepared AuNPs that have a characteristic band at 524 nm, corresponding to a 20-nm particle size. To confirm the particular size and shape, synthesized AuNPs were further analyzed using TEM.

### TEM analysis

**Figure 6:**
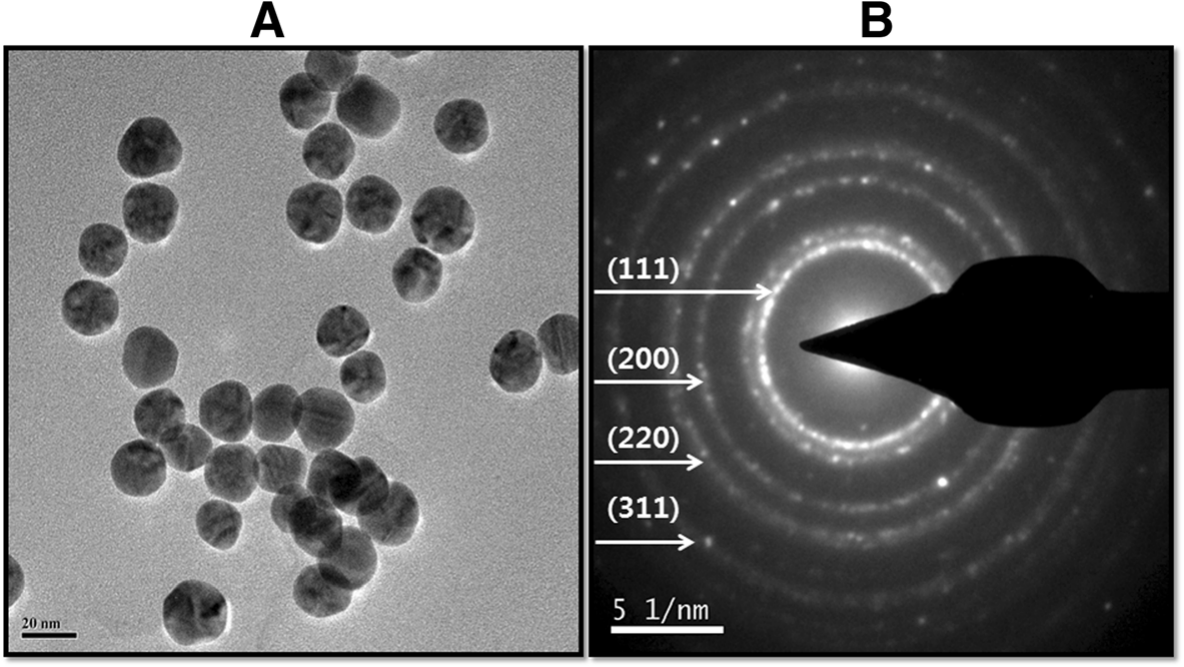
**Size and shape analysis of AuNPs by TEM.** Several fields were photographed and used to determine the size and morphology of AuNPs **(A)**. Selected area of electron diffraction pattern **(B)**.

TEM micrographs of the AuNPs revealed distinct, uniform molecules that were spherical in shape and well separated from each other (Figure 6). The average particle size was estimated from counting more than 200 particles from TEM images, and the average size of homogeneous, spherical AuNPs was 20 nm. Interestingly, the AuNPs synthesized by *Ganoderma* spp. are spherical and smaller than those synthesized by other fungi, such as *Colletotrichum* spp. [51] and edible mushrooms [32]; most importantly, the prepared nanoparticles were homogeneous and spherical in shape.

Homogeneous nanoparticles with specific shapes are important for applications in biological and chemical sensing as well as for optical, medical, and electronic devices because the optical properties of AuNPs are dependent on the size and shape [56]. Several studies have reported synthesis of various size AuNPs using different fungi. *Fusarium oxysporum* produced spherical and triangular morphologies of particles with a size range of 20 to 40 nm [15]. Honary et al. [57] reported that *Penicillium aurantiogriseum*, *Penicillium citrinum*, and *Penicillium waksmanii* synthesized AuNPs that were fairly uniform with spherical shapes and had average diameters of 153.3, 172, and 160.1 nm, respectively. Alternatively, the fungi *Aspergillus fumigates*[30] and *Neurospora crassa*[36] produced average AuNP sizes of 25 and 32 nm, respectively.

### Effect of AuNPs on cell viability

The use of nontoxic and biocompatible nanoparticles with capping materials is an important aspect of biomedical applications. Consequently, the cytotoxic effects and future health problems caused by nanoparticles must be considered in the engineering of such materials. It is essential to validate whether as-prepared AuNPs are toxic or biocompatible, because biomedical applications of any nanomaterial involves intentional exposure to nanoparticles. Therefore, understanding the properties of nanoparticles and their effects on the human body are crucial before they are clinically applied [58].

**Figure 7:**
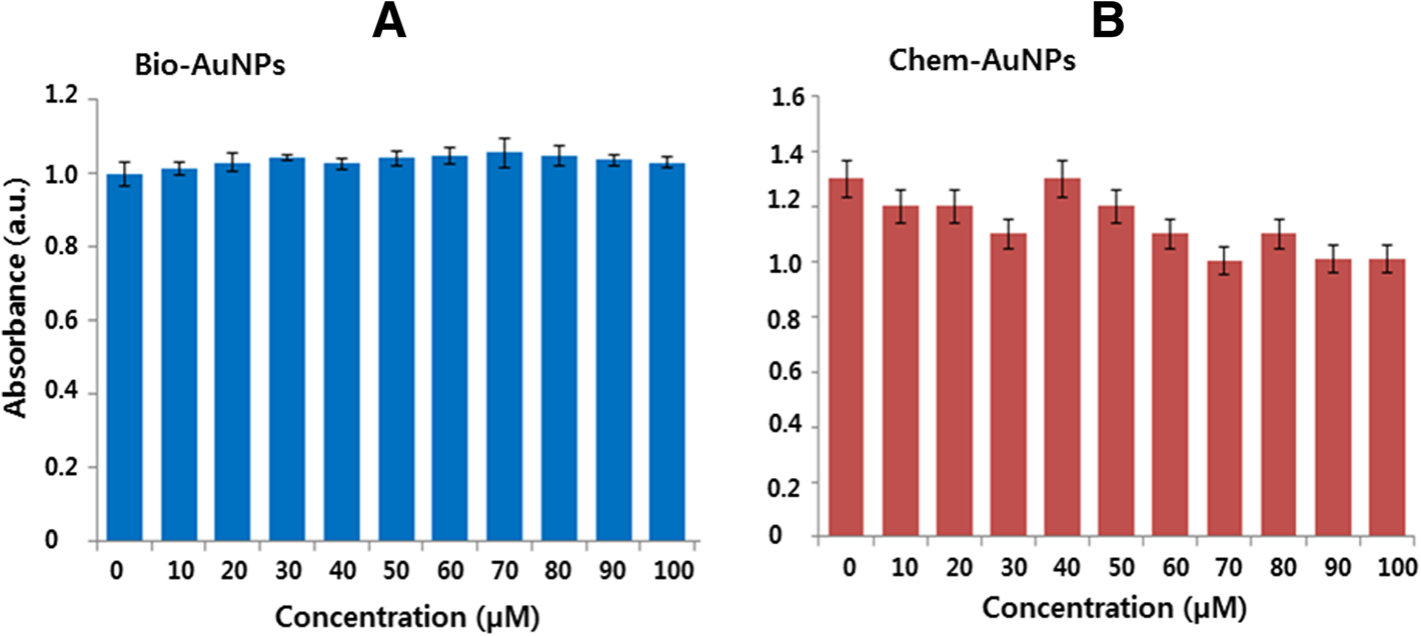
**The effect of AuNPs on cell viability of MDA-MB-231 human breast cancer cells.** MDA-MB-231 human breast cancer cells were treated with bio-AuNPs **(A)** or chem-AuNPs **(B)** at various concentrations from 0 to 100 μM/mL for 24 h, and cell viability was determined by the MTT method. The results are expressed as the mean ± SD of three separate experiments, each of which contained three replicates. Treated groups were not statistically different from the control group based on the Student's *t* test.

The biocompatibility of both AuNPs was assessed by a proliferation assay, using mitochondrial functional activity as an indicator of cell viability. The cells were treated with different concentrations of both bio- and chem-AuNPs for 24 h, using the cell viability assay. We found that cells treated with bio-AuNPs showed no toxic effect, even at higher concentrations (Figure 7A), which suggests that as-prepared AuNPs possess biocompatible properties, whereas the chem-AuNPs showed a slight decrease of cell viability at higher concentrations. However, the results are not statistically different from those of the controls. It was also confirmed by incubating AuNPs with medium only and checking the absorption at a wavelength used for MTT assay that the presence of all tested AuNPs did not interfere with the assay.

Shukla et al. [59] suggested that AuNPs are not cytotoxic, reduce the production of reactive oxygen and nitrite species, and do not stimulate secretion of proinflammatory cytokines, such as TNF-alpha and IL1-beta, making them suitable candidates for nanomedicine. Using a human leukaemia cell line, gold nanospheres of different sizes (4, 12, and 18 nm in diameter) and capping agents were found to be nontoxic based on the MTT assay [60]. Similarly, Arnida et al. [61] found that plain spherical AuNPs and PEGylated spheres and rods did not interfere with the proliferation of PC-3 cells when cells were exposed to as high as 1.5 nM of AuNPs for a period of over two population doubling times (88 h). Plain spherical particles that were 50 and 90 nm in diameter slightly stimulated the proliferation of PC-3 cells. Parab et al. [58] investigated the biocompatibility effect of sodium hexametaphosphate (HMP)-stabilized AuNPs (Au-HMPs) in tumor and fibroblast cells. Synthesized Au-HMP nanoparticles and their surface-modified counterparts revealed non-cytotoxic properties in tested cells and showed biocompatibility.

Mukherjee et al. [38] designed and developed an AuNP-based drug delivery system (DDS) (Au-DOX) containing doxorubicin (DOX). Administration of this DDS to breast cancer cells (MCF-7 and MDA-MB-231) showed significant inhibition of breast cancer cell proliferation compared with pristine doxorubicin. The viability of the bovine retinal pigment epithelial cells was not affected with an AuNP concentration of up to 300 nM, and increasing the concentrations above 300 nM resulted in significant cell death [62]. AuNPs have anti-oxidative and anti-hyperglycemic activities in streptozotocin-induced diabetic mice by balancing or inhibiting ROS generation in hyperglycemic conditions by scavenging free radicals and leading to increased anti-oxidant defense enzymes in mice.

### Effect of AuNPs on LDH leakage

**Figure 8:**
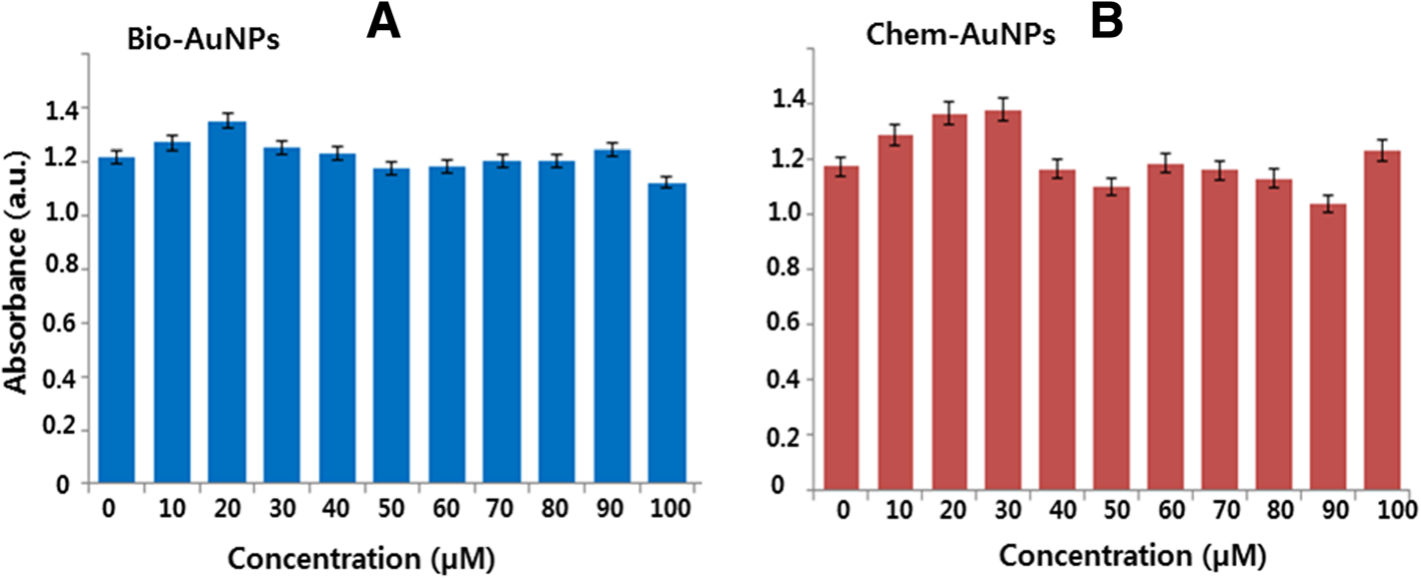
**The effect of AuNPs on membrane integrity of cells.** MDA-MB-231 human breast cancer cells were treated with bio-AuNPs **(A)** or chem-AuNPs **(B)** at various concentrations from 0 to 100 μM/mL for 24 h, and LDH leakage was estimated as described in the ‘Methods’ section. The results are expressed as the mean ± SD of three separate experiments, each of which contained three replicates. Treated groups were not statistically different from the control group based on the Student's *t* test (*p* > 0.05).

To determine whether AuNPs have an effect on LDH leakage, which is a vital marker of cell death, the cells were treated with various concentrations of bio- and chem-AuNPs (0 to 100 μM) for 24 h. It is interesting to observe that, when treated with both AuNP solutions, there was no change in the leakage of LDH level for up to 24 h in comparison with the untreated control (Figure 8). The LDH and cell viability data are consistent and show no reduction of cell proliferation at higher gold concentrations after 24 h.

Pan et al. [63] found that 1.4-nm gold nanospheres triggered necrosis and mitochondrial damage and induced oxidative stress in endothelial and epithelial cells. In contrast, they found no evidence of cellular damage for 15-nm gold nanospheres bearing the same surface group [63], and these results also suggest that the toxicity of AuNPs depends on size. Interestingly, citrate-capped AuNPs (13 nm in diameter) were found to be toxic to a human carcinoma lung cell line but not to a human liver carcinoma cell line at the same dosage [64].

Uboldi et al. [65] reported that, after 24 to 48 h of exposure, AuNPs induced a mild LDH release in the human ATII-like cell line A549, independent of the presence or absence of surface contaminants. Additionally, after 72 h of exposure to AuNPs, there was a dose-dependent release of LDH in the supernatant, and the amount of LDH released was significantly higher compared with shorter exposure times. Zhang et al. [66] reported that chloroplast-mediated synthesis of AuNPs retained up to 85% better viability in both GES-1 and MGC-803 cells, even up to 150 μg/mL after 36 h of treatment. Freese et al. [67] studied the effect of AuNPs on the amount of LDH released into the supernatant, and they suggest that up to 100 μM of AuNPs did not induce cytotoxicity in human dermal microvascular endothelial cells (HDMECs) and human cardiac microvascular endothelial cells (hCMECs). Altogether, our findings suggest that the biologically derived AuNPs with an average size of 20 nm are biocompatible.

### ROS generation

ROS, which are a specific type of oxygen-containing reactive molecule, play important roles in various cellular processes and are known to be essential for basal cell proliferation [68]. Higher concentrations of ROS lead to cell death [69,70]. Several studies suggested that nanoparticle-mediated cytotoxicity is associated with ROS production.

**Figure 9:**
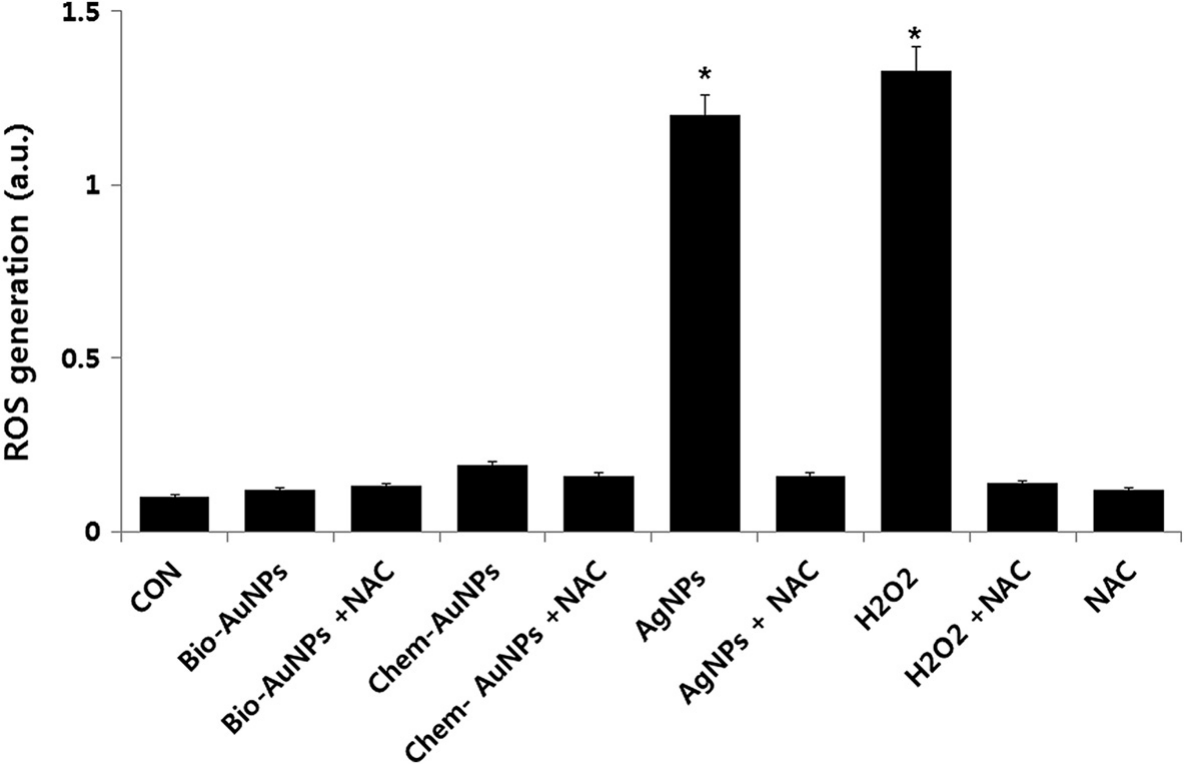
**The effect of AuNPs in ROS generation.** Relative fluorescence of DCF was measured using a spectrofluorometer with excitation at 485 nm and emission at 530 nm. The results are expressed as the mean ± SD of three separate experiments, each of which contained three replicates. Treated groups with bio- and chem-AuNPs were not statistically different from the control group based on the Student's *t* test. (*p* > 0.05). H_2_O_2_- and AgNP-treated groups were statistically different from the control group based on the Student's *t* test (*p < 0.05).

In this case, we further examined the effect of AuNPs on oxidative stress utilizing the fluorescent dye H_2_DCFDA, which does not exhibit enhanced fluorescence in the presence of AuNPs. Results from exposure of MDA-MB-231 to 100 μM of AuNP solution indicate that there are no noticeable increases of ROS generation at the treated concentrations. The results indicate that both bio- and chem-AuNPs are largely ineffective at inducing ROS generation in MDA-MB-231 cells, whereas H_2_O_2_- and AgNP-treated groups showed remarkable increase in ROS generation (Figure 9).

Chuang et al. [71] extensively studied the exposure of three different-sized AuNPs in human gastric carcinoma (AGS) and human lung adenocarcinoma epithelial (A549) cells. Their results suggest that significant increases of ROS generation occur with certain concentrations of AuNPs in AGS cells. Conversely, no obvious increases were observed for A549 cells in any of the three sizes of AuNPs. The authors eventually concluded that ROS signaling may play a role in AuNP-induced apoptotic cell death in AGS cells.

Furthermore, western blot analyses revealed that the expression of proteins involved in the anti-oxidative defense system was not significantly modulated any of the three sizes of AuNPs in both lines, except for a modest increase in TrxR-1 and SOD-1 in AGS cells [71]. Altogether, our results suggest that biologically synthesized AuNPs have significant biocompatibility and could possibly be used for ultrasensitive detection, gene transfer, biomolecular imaging, drug delivery, and cancer therapy.

## Conclusion

Synthesis of nanoparticles using biological systems is an important area of nanobiotechnology. Here we show a simple, rapid, clean, efficient, cost-effective, and green method for the synthesis of biocompatible AuNPs using *Ganoderma* spp*.* extract as a reducing and stabilizing agent. The as-prepared AuNPs were characterized *via* UV-vis, XRD, FTIR, EDX, DLS, and TEM. The biologically derived AuNPs were spherical, discrete, and the average size was 20 nm. The biocompatibility effect of AuNPs was investigated using cell viability, LDH, and ROS assays. The results indicate that biologically derived AuNPs are biocompatible. Finally, this eco-friendly method provides an alternative route for large-scale production of biocompatible AuNPs that can be used in catalysis, sensors, electronics, and biomedical applications, especially for cancer therapy.

## Competing interests

The authors declare that they have no competing interests.

## Authors' contributions

SG came up with the idea and participated in the design, preparation of AuNPs, and writing of the manuscript. JWH performed characterization of nanoparticles. JHP participated in culturing, cell viability, LDH, and ROS assay. SG and JHK participated in coordination of this study. All authors read and approved the final manuscript.
